# Low-Pass Filtering Empirical Wavelet Transform Machine Learning Based Fault Diagnosis for Combined Fault of Wind Turbines

**DOI:** 10.3390/e23080975

**Published:** 2021-07-29

**Authors:** Yancai Xiao, Jinyu Xue, Mengdi Li, Wei Yang

**Affiliations:** School of Mechanical, Electronic and Control Engineering, Beijing Jiaotong University, Beijing 100044, China; 20126082@bjtu.edu.cn (J.X.); 18121281@bjtu.edu.cn (M.L.); 19116025@bjtu.edu.cn (W.Y.)

**Keywords:** combined fault diagnosis, empirical wavelet transform, grey wolf optimizer, low pass FIR filter, support vector machine

## Abstract

Fault diagnosis of wind turbines is of great importance to reduce operating and maintenance costs of wind farms. At present, most wind turbine fault diagnosis methods are focused on single faults, and the methods for combined faults usually depend on inefficient manual analysis. Filling the gap, this paper proposes a low-pass filtering empirical wavelet transform (LPFEWT) machine learning based fault diagnosis method for combined fault of wind turbines, which can identify the fault type of wind turbines simply and efficiently without human experience and with low computation costs. In this method, low-pass filtering empirical wavelet transform is proposed to extract fault features from vibration signals, LPFEWT energies are selected to be the inputs of the fault diagnosis model, a grey wolf optimizer hyperparameter tuned support vector machine (SVM) is employed for fault diagnosis. The method is verified on a wind turbine test rig that can simulate shaft misalignment and broken gear tooth faulty conditions. Compared with other models, the proposed model has superiority for this classification problem.

## 1. Introduction

With the improvement of people’s environmental awareness, sustainable and carbon-neutral renewable energy has gradually developed to replace oil, coal and other traditional fossil fuels [1]. According to a recent report about renewable capacity statistics [2], the world’s wind energy capacity is 622,704 MW in 2019, accounting for 24.55% of the total renewable energy capacity, second only to the hydropower which is the oldest renewable energy source [3]. The annual growth rate of wind energy is 10.44% in 2019, second only to the rapidly developing solar energy. Improving the efficiency of wind turbines has always been a hot issue in terms of wind energy utilization. In addition to study the selection of wind turbine [4,5,6], it is useful to reasonably design the wind turbines’ structure [7,8]. At the same time, wind turbines are usually exposed to dynamic and harsh weather conditions, experiencing variable and rough working environments, which makes them prone to failure than other ordinary machinery. If a component of the wind turbine is broken without awareness of workers, it may well cause damage to other components, and even lead to the shutdown of the wind turbine, resulting in huge economic losses [9]. Operating and maintenance costs account for more than 25% of total costs for onshore wind farms and these costs are even higher for offshore projects [10]. Therefore, it is of great significance to reduce maintenance costs and improve the efficiency of wind farms by detecting the fault of wind turbines in time.

Many studies have been carried out on fault diagnosis of wind turbines. Such as Liu et al. [11] introduced local mean decomposition (LMD) to analyze the wind turbine gearbox vibration signals for fault diagnosis. Feng et al. [12] proposed a frequency demodulation analysis method based on the ensemble empirical mode decomposition (EEMD) and energy separation algorithm to detect and locate the fault of wind turbine planetary gearbox by analyzing vibration signals. Chen et al. [13] applied empirical wavelet transformation (EWT) to vibration signals to diagnose wind turbine generator bearings faults. Those methods depend on experienced people to analyze the signal and determine the fault of drivetrains of wind turbines, although the precision is guaranteed, it is lack of efficiency. In recent years, with the rise of machine learning (ML), some scholars have tried to use ML methods to diagnosis the drivetrain of wind turbines. For example, Liu et al. [14] extracted features from vibration signals by diagonal spectrum and employed clustering binary tree support vector machines to diagnosis the wind turbines gearbox. Tang et al. [15] proposed a fault diagnosis method for the drivetrain of wind turbines based on manifold learning and Shannon wavelet support vector machine. Gao et al. [16] decomposed vibration signals by integral extension local mean decomposition (IELMD) and calculated multiscale entropy values as features for least squares support vector machines to identify fault type of rolling bearing in wind turbine gearbox. Lei et al. [17] introduced long-short term memory (LSTM) networks in wind turbine fault diagnosis. Jiang et al. [18] proposed multiscale convolutional neural network (MSCNN) to diagnose wind turbine gearbox faults.

Almost two-thirds of ML-based wind turbine fault diagnosis methods use classification, whose procedures include preprocess data, equalize classes, feature extraction, feature selection, hyperparameter tuning, cross-validation and use the best model [19]. This intelligent way allows the diagnosis to be free from expert experience.

However, most of these ML-based wind turbine fault diagnosis methods only studied on single fault [15,16,17,18,19]. In reality, a wind turbine is a complex system, failures could happen one after another or simultaneously, therefore, a wind turbine may have more than one fault at the same time, i.e., combined fault occurs. For example, misalignment may lead to gear or bearing fails, then multiple faults coexist. Gear faults in different stages is also a common combined fault [20]. Combined fault (also called compound fault) is more difficult to diagnose than single fault because typical fault features will become difficult to be extracted. At present, combined fault diagnosis of wind turbines usually depends on manual analysis to calculate, extract and show the frequencies of different faults in spectrums [21,22,23,24,25,26,27]. Only a few scholars have studied combined fault diagnosis by ML. For example, Zhong et al. [28] decomposed the vibration signal into a series of intrinsic mode functions (IMFs) by Hilbert-Huang transform (HHT) with ensemble empirical mode decomposition (EEMD), then selected useful IMFs by correlation coefficients, and calculated the energy vector from the selected IMFs together with maximum amplitude and corresponding frequency and six time-domain statistical indices as features of pairwise-coupled sparse Bayes extreme learning machine to detect several common gearbox single-faults and simultaneous-faults.

This paper will focus on a ML-based fault diagnosis method for combined faults and single faults of wind turbines. In our method, a composite fault is considered as a fault equivalent to a single fault, which means the output of a combined fault is not multiple binary tags for each single fault (multilabel classification problem). The reminder of this paper is structured as follows: Section 2 introduces the proposed method and related theories. Section 3 presents the test rig, the experiments and the results. Finally, the conclusion in Section 4.

## 2. Methods

**Figure 1:**
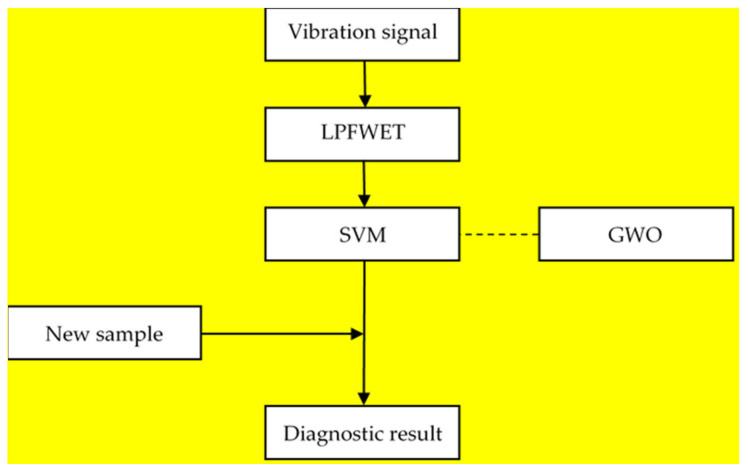
The flow chart of the proposed ML-based fault diagnosis method for combined fault of wind turbines.

The fault diagnosis method for combined fault of wind turbines we proposed can be described as follows. First, extract features from vibration signals by low pass filtering empirical wavelet transform (LPFEWT). Then, build features datasets in different conditions (normal, single faults and combined fault). Last, train the support vector machine (SVM) for classification, using grey wolf optimizer (GWO) for hyperparameter tuning. After training, the obtained SVM model can identify faults of wind turbines by inputting features of vibration signals. The flow chart of the method is shown in Figure 1.

### 2.1. Low Pass Filtering Empirical Wavelet Transform (LPFEWT)

Empirical Wavelet Transform (EWT) is a new adaptive signal processing approach proposed by Gilles in 2013 [29]. The main idea is to adaptively decompose the modes of a signal from its Fourier spectrum by an appropriately built wavelet filter bank. The steps of EWT are summarized as follows:

- Fast Fourier Transform (FFT);

Convert the signal *f* to the frequency domain by FFT to get its Fourier spectrum (frequency $\omega \in \;\left[0,\pi \right]$).

- Fourier Spectrum Segmentation;

Divide the Fourier spectrum into *N* contiguous segments. Let *ϖ_n_* denote the limits between each segment. Each segment is denoted as $\Lambda_{n}=\;\left[\omega_{n-1},\omega_{n}\right]$. With each *ϖ_n_* as center, a transition phase of width $2\tau_{n}$ is defined.

- Mode Extraction;

Let $\hat{f}$ and $\overset{ˇ}{f}$ denote the Fourier transform and its inverse respectively. Choose *τ_n_* proportional to *ϖ_n_*: $\tau_{n}=\gamma \omega_{n}$, where 0<γ<1. Consequently, ∀n>0, the empirical scaling function ${\hat{\phi }}_{n}\left(\omega \right)$ and the empirical wavelets ${\hat{\psi }}_{n}\left(\omega \right)$ are as follows:

(1) $${\hat{\phi }}_{n}\left(\omega \right)=\left\{\begin{array}{l} 1,\;\;\;\;\left|\omega \right|\leq \left(1-\gamma \right)\omega_{n}\\ cos\left[\frac{\pi }{2}\beta \left(\frac{1}{2\tau_{n}}\;\left(\left|\omega \right|-\left(1-\gamma \right)\omega_{n}\right)\right)\right],\\ \;\;\;\;\;\;\left(1-\gamma \right)\omega_{n}\leq \left|\omega \right|\leq \left(1+\gamma \right)\omega_{n}\\ 0,\;\;otherwise \end{array}\right.$$

and

(2) $${\hat{\psi }}_{n}\left(\omega \right)=\left\{\begin{array}{l} 1,\;\;\;\;\omega_{n}+\tau_{n}\leq \left|\omega \right|\leq \left(1-\gamma \right)\omega_{n+1}\\ cos\left[\frac{\pi }{2}\beta \left(\frac{1}{2\tau \omega_{n+1}}\;\left(\left|\omega \right|-\left(1-\gamma \right)\omega_{n+1}\right)\right)\right],\\ \;\;\;\;\;\;\left(1-\gamma \right)\omega_{n+1}\leq \left|\omega \right|\leq \left(1+\gamma \right)\omega_{n+1}\\ sin\left[\frac{\pi }{2}\beta \left(\frac{1}{2\gamma \omega_{n}}\;\left(\left|\omega \right|-\left(1-\gamma \right)\omega_{n}\right)\right)\right],\\ \;\;\;\;\;\;\left(1-\gamma \right)\omega_{n}\leq \left|\omega \right|\leq \left(1+\gamma \right)\omega_{n}\\ 0,\;\;otherwise \end{array}\right.$$

To construct a tight frame set of empirical wavelets, choose

(3) $$\gamma <{min}_{n}\left(\frac{\omega_{n+1}-\omega_{n}}{\omega_{n+1}+\omega_{n}}\right)$$

The detail coefficients $W_{f}^{\varepsilon }\left(n,t\right)$ are given by the inner products with the empirical wavelets function ${\hat{\psi }}_{n}\left(\omega \right)$, and the approximation coefficients $W_{f}^{\varepsilon }\left(0,t\right)$ are given by the inner product with the scaling function ${\hat{\phi }}_{1}\left(\omega \right)$.

(4) $$\begin{array}{ll} W_{f}^{\varepsilon }\left(n,t\right) & =\langle f,\psi_{n}\rangle =\int f\left(\tau \right)\;\bar{\psi_{n}\left(\tau -t\right)}d\tau \\ & ={\left(\hat{f}\left(\omega \right)\bar{\psi_{n}\left(\omega \right)}\right)}^{ˇ} \end{array}$$

(5) $$\begin{array}{ll} W_{f}^{\varepsilon }\left(0,t\right) & =\langle f,\phi_{1}\rangle =\int f\left(\tau \right)\;\bar{\phi_{1}\left(\tau -t\right)}d\tau \\ & ={\left(\hat{f}\left(\omega \right)\bar{\phi_{1}\left(\omega \right)}\right)}^{ˇ} \end{array}$$

The reconstruction is obtained by

(6) $$\begin{array}{ll} f\left(t\right) & =W_{f}^{\varepsilon }\left(0,t\right)⋆\phi_{1}\left(t\right)+\overset{N}{\underset{n=1}{\sum }}W_{f}^{\varepsilon }\left(n,t\right)⋆\psi_{n}\left(t\right)\\ & ={\left(\hat{W_{f}^{\varepsilon }}\left(0,\omega \right)\hat{\phi_{1}}\left(\omega \right)+\overset{N}{\underset{n=1}{\sum }}\hat{W_{f}^{\varepsilon }}\left(n,t\right)\hat{\psi_{n}}\left(t\right)\right)}^{ˇ} \end{array}$$

There are multiple algorithms to automatically segment the Fourier spectrum, such as local-maxima, local-maxima-minima and scale-space (including otsu, half-normal, empirical law, means and k-means) [29,30]. The scale-space algorithms are parameterless, but it takes long time for the computation when processing a long signal. And different signals are often decomposed into different amounts of modes, which is inconvenient for the comparison with each other. Considering these factors, we choose the simplest and fastest algorithm–local-maxima, which can set the max number of segments.

Based on EWT, LPFEWT is proposed to extract features. First, design a low pass FIR filter with an appropriate cut-off frequency for the signal. Next, employ EWT on the filtered signal to decompose the signal into several empirical modes. Then, exclude the empirical mode of the highest frequencies which is mostly affected by the filter. Last, calculate the indices of the left modes as features. According to this approach, the feature required for fault diagnosis can be obtained easily.

Compared to the tradition wavelet transform, LPFEWT is adaptive, which means it decomposes the signal based on the information contained in the signal itself so that there is no need to choose or design specific wavelet basis for the signal.

### 2.2. Support Vector Machine (SVM)

SVM is a very powerful and versatile ML model and particularly well suited for classification of complex but small- or medium-sized datasets [31].

The simplest linear SVM for binary classification can be described as follows. For all samples to be classified $x_{i}\left(i=1,2,\ldots ,m\right)$, the output is

(7) $$y_{i}=sign\left(w^{T}x_{i}+b\right)$$

i.e., $y_{i}=-1$ if $w^{T}x_{i}+b<0$, $y_{i}=+1$ if $w^{T}x_{i}+b>0$. So the hyperplane $w^{T}x+b=0$ is decision boundary. To make the decision boundary best for separation, construct two hyperplanes $w^{T}x+b=-1$ and $w^{T}x+b=1$ which are parallel and at equal distance to the decision boundary, i.e., $y_{i}=-1$ if $w^{T}x_{i}+b\leq -1$, $y_{i}=+1$ if $w^{T}x_{i}+b\geq 1$. Training SVM means finding the value of w and b that make the width of the margin 2/∥w∥ as large as possible. That is a constrained optimization problem

(8) $$\underset{w,b}{max}\frac{2}{∥w∥}\;\;\;\;s.t.\;\;y_{i}\left(w^{T}x_{i}+b\right)\geq 1,\;i=1,2,\ldots ,n$$

which can be converted to an equivalent problem

(9) $$\underset{w,b}{min}\frac{1}{2}{∥w∥}^{2}\;\;\;\;s.t.\;\;y_{i}\left(w^{T}x_{i}+b\right)\geq 1,\;i=1,2,\ldots ,n$$

This is a convex quadratic optimization problem with linear constraints, which is known as quadratic programming (QP) problems and can be solved by the method of Lagrange multipliers. Introduce Lagrange multipliers $\lambda =\left(\lambda_{1},\lambda_{2},\cdots ,\lambda_{m}\right)$, the objective function of optimization can be expressed as

(10) $$\begin{array}{c} L\left(w,b,\lambda \right)=\frac{1}{2}{∥w∥}^{2}+\overset{n}{\underset{i=1}{\sum }}\lambda_{i}\left[1-y_{i}\left(w^{T}x_{i}+b\right)\right]\\ \lambda_{i}\geq 0,i=1,2,\cdots ,n\;\;\; \end{array}$$

The problem is to solve

(11) $$\underset{w,b}{min}\;\underset{\lambda }{max}\;L\left(w,b,\lambda \right)$$

The dual problem is

(12) $$\underset{\lambda }{max}\;\underset{w,\;b}{min}\;L\left(w,b,\lambda \right)$$

Calculate the gradients of both w and b, and set them equal to zero.

(13) $$\nabla_{w}L\left(w,b,\lambda \right)=w-\overset{n}{\underset{i=1}{\sum }}\lambda_{i}x_{i}y_{i}=0\;$$

(14) $$\frac{\partial }{\partial b}L\left(w,b,\lambda \right)=-\overset{n}{\underset{i=1}{\sum }}\lambda_{i}y_{i}=0$$

Substitute (13) and (14) into problem (12), obtain

(15) $$\begin{array}{c} \underset{\lambda }{max}\overset{n}{\underset{i=1}{\sum }}\lambda_{i}-\frac{1}{2}\overset{n}{\underset{i=1}{\sum }}\overset{n}{\underset{j=1}{\sum }}\lambda_{i}\lambda_{j}y_{i}y_{j}(x_{i}\cdot x_{j})\\ s.t.\;\overset{n}{\underset{i=1}{\sum }}\lambda_{i}y_{i}=0,\lambda_{i}\geq 0,i=1,2,\ldots ,m\;\;\;\;\;\;\;\;\;\;\;\;\;\;\;\;\;\;\;\;\;\;\;\;\;\;\;\;\;\;\;\; \end{array}$$

Consequently, the original minimization problem about w and b is converted to a QP problem about solving λ.

To make the model more flexible, soft margin classification is proposed which allows few instances between the margins or even on the wrong side. Soft margin SVM introduces slack variable $\xi_{i}\left(i=1,2,\cdots ,n\right)$, so the problem becomes

(16) $$\begin{array}{c} \underset{w,b,\xi }{min}\frac{1}{2}{∥w∥}^{2}+C\overset{n}{\underset{t=1}{\sum }}\xi_{i}\\ s.t.\;\;y_{i}\left(w^{T}x_{i}+b\right)\geq 1-\xi_{i},\;\xi_{i}>0,\;i=1,2,\ldots ,m \end{array}$$

where C is penalty term. The bigger the C, the more penalty SVM gets when it makes misclassification, the less the tolerance, the smaller the margin.

The QP problem equivalent to soft margin SVM classification is

(17) $$\begin{array}{c} \underset{\lambda }{max}\overset{n}{\underset{i=1}{\sum }}\lambda_{i}-\frac{1}{2}\overset{n}{\underset{i=1}{\sum }}\overset{n}{\underset{j=1}{\sum }}\lambda_{i}\lambda_{j}y_{i}y_{j}(x_{i}\cdot x_{j})\\ s.t.\;\overset{n}{\underset{i=1}{\sum }}\lambda_{i}y_{i}=0,0\leq \lambda_{i}\leq C,i=1,2,\ldots ,m\;\;\;\;\;\;\;\;\;\;\;\; \end{array}$$

For problems that are not linearly separable, transformation ϕ is introduced to map x from the original space to a higher dimensional space $\phi \left(x\right)$, which makes it easier to find a linear decision boundary in the new feature space. The kernel function $K\left(x_{i},x_{j}\right)=\phi \left(x_{i}\right)\cdot \;\phi \left(x_{j}\right)$ is proposed to focus on the results without computing the coordinates of the data in the new space. The kernel trick makes the whole process much more computationally efficient. Problem (17) can be rewritten as

(18) $$\begin{array}{c} \underset{\lambda }{max}\overset{n}{\underset{i=1}{\sum }}\lambda_{i}-\frac{1}{2}\overset{n}{\underset{i=1}{\sum }}\overset{n}{\underset{j=1}{\sum }}\lambda_{i}\lambda_{j}y_{i}y_{j}K\left(x_{i},x_{j}\right)\\ s.t.\;\overset{n}{\underset{i=1}{\sum }}\lambda_{i}y_{i}=0,0\leq \lambda_{i}\leq C,i=1,2,\ldots ,m\;\;\;\;\;\;\;\;\;\;\;\; \end{array}$$

In this paper, we use radial basis function (RBF) kernel as below

(19) $$K\left(x_{i},x_{j}\right)=e^{-\gamma ∥x_{i}-x_{j}∥{}^{2}},\gamma >0$$

RBF kernel is one of the most used kernel functions, which can deal with both linear and nonlinear classification problems. The result of linear classification using RBF kernel is comparable to using linear kernel [32,33].

### 2.3. Grey Wolf Optimizer

**Figure 2:**
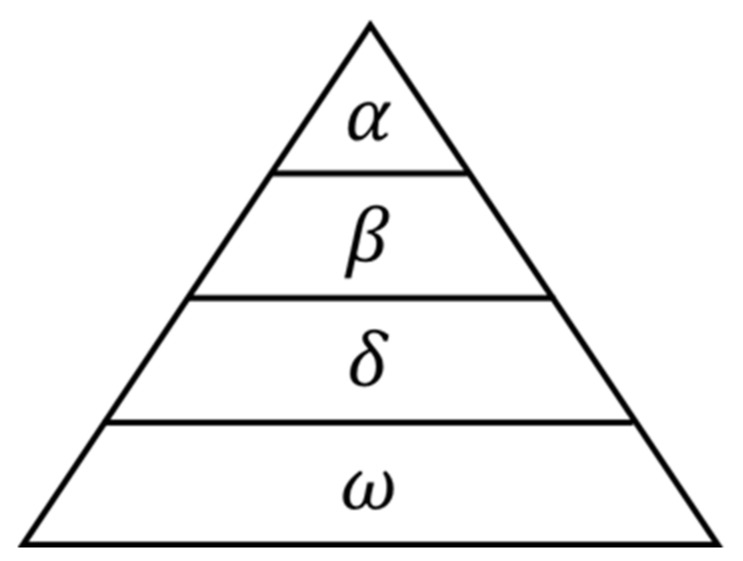
The social hierarchy of grey wolves.

Grey Wolf Optimizer (GWO) is a swarm intelligence (SI) algorithm proposed by Mirjalili et al. [34] in 2014 that imitates the leadership hierarchy and hunting mechanism of grey wolves in nature. In this paper, it is used to optimize the parameters in SVM. The social hierarchy of gray wolves is shown in Figure 2. Grey wolves are divided into four levels from α to ω. The upper level wolves dominate the lower level ones, and the lower level wolves follow the upper level ones.

In the GWO algorithm, imitating the social hierarchy of grey wolves, the first best candidate solution is regarded as α, the second best candidate solution is regarded as β, the third best candidate solution is regarded as δ, the remaining candidate solutions are regarded as ω. The hunting (optimization) is guided by α, β and δ, while ω follow them. The encircling behavior is modeled as follows:

(20) $$D=\left|C\;\cdot X_{p}\left(t\right)-X\left(t\right)\right|$$

(21) $$X\left(t+1\right)=X_{p}\left(t\right)-A\cdot D$$

where t represents the number of iterations, A and C are coefficient vectors, $X_{p}$ is the position vector of the prey (optimum), X is the position vector of a grey wolf, and D represents the distance between the grey wolf and the prey.

The vectors A and C are defined as follows:

(22) $$A=2a\cdot r_{1}-a$$

(23) $$C=2\cdot r_{2}$$

where components of a are linearly dropped from 2 to 0 over the course of iterations, components of $r_{1}$ and $r_{2}$ are random numbers in $\left[0,1\right]$.

The random vectors $r_{1}$ and $r_{2}$ allow grey wolves to move any position within a certain range of the prey. With the vector a decreases, grey wolves encircle and pursue the prey. The location of the prey is replaced by the decisions of all three grey wolves α, β and δ. The following equations are used for updating the position of each grey wolf.

(24) $$\left\{\begin{array}{c} D_{\alpha }=\left|C_{1}\cdot X_{\alpha }-X\left(t\right)\right|\\ D_{\beta }=\left|C_{2}\cdot X_{\beta }-X\left(t\right)\right|\\ D_{\delta }=\left|C_{3}\cdot X_{\delta }-X\left(t\right)\right| \end{array}\right.$$

(25) $$\left\{\begin{array}{c} X_{1}=X_{\alpha }-A_{1}\cdot D_{\alpha }\\ X_{2}=X_{\beta }-A_{2}\cdot D_{\beta }\\ X_{3}=X_{\delta }-A_{3}\cdot D_{\delta } \end{array}\right.$$

(26) $$X\left(t+1\right)=\frac{X_{1}+X_{2}+X_{3}}{3}$$

Since A is a random vector in the interval $\left[-a,a\right]$, the next position of wolves will approach the prey if $\left|A\right|<1$, and move away from the prey if $\left|A\right|>1$. This means that grey wolves not only pursue and attack current prey but also leave to search for other prey. In other words, the GWO algorithm has exploration feature to help avoid local optima. The random vector C simulates the obstacles to approaching prey in nature.

GWO can make the process of hyperparameter tuning of SVM more effective than normal way (grid search or randomized search). Also, GWO hyperparameter tuned has better classification accuracy than the typical one-versus-one multi-class SVM [35]. Compared with particle swarm optimization (PSO), GWO has fewer parameters to be determined, only the population and the max number of iterations, because it updates the positions of search agents by the positions of the three best wolves, while PSO updates the positions of search agents by the global best position and the personal best position, and each search agent has velocity besides position.

## 3. Experimental Results

### 3.1. Experimental Test Rig and Data Collection

**Figure 3:**
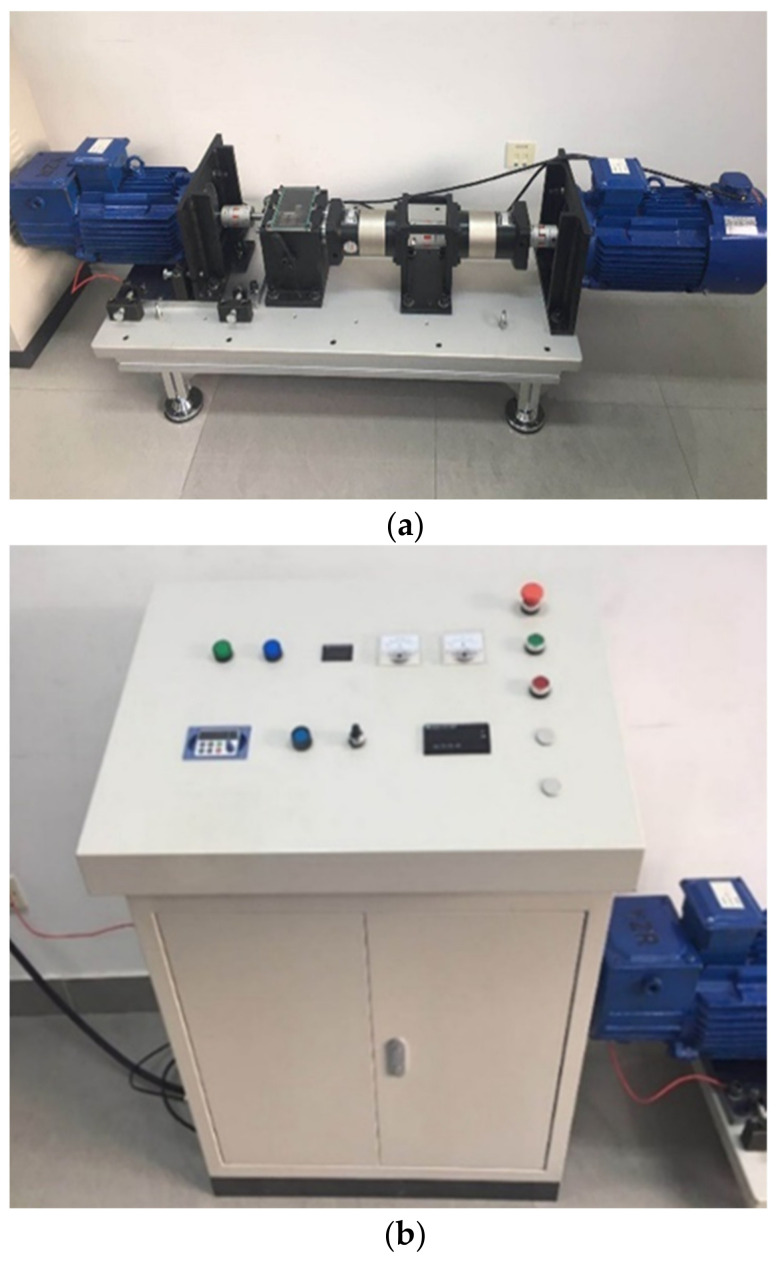
Wind turbine drivetrain fault experimental test rig: (**a**) experimental test bench; (**b**) control panel cabine.

The laboratory’s wind turbine drivetrain fault test rig is shown in Figure 3, which consists of a control panel cabinet and an experimental test bench to simulate doubly-fed induction generator (DFIG) wind turbine shaft misalignment (between the gearbox and the generator) and broken gear tooth faulty conditions. In Figure 3a, the speed of the motor of the experimental test bench on the right side is decelerated by a planetary gear reducer to simulate the wind blowing blade speed, then it is accelerated by a planetary gear accelerator and a gearbox to drive the generator. The maximum speed of the driving motor is 720 r/min, the speed of the generator is 500 r/min. The left gearbox can be adjusted by the handle to select a normal gear or a broken gear. The generator can be adjusted by the support to create offset or angular misalignment. The control panel cabinet shown in Figure 3b can set and display the motor speed, showing the angle between the generator and the gearbox and other electrical parameters.

**Figure 4:**
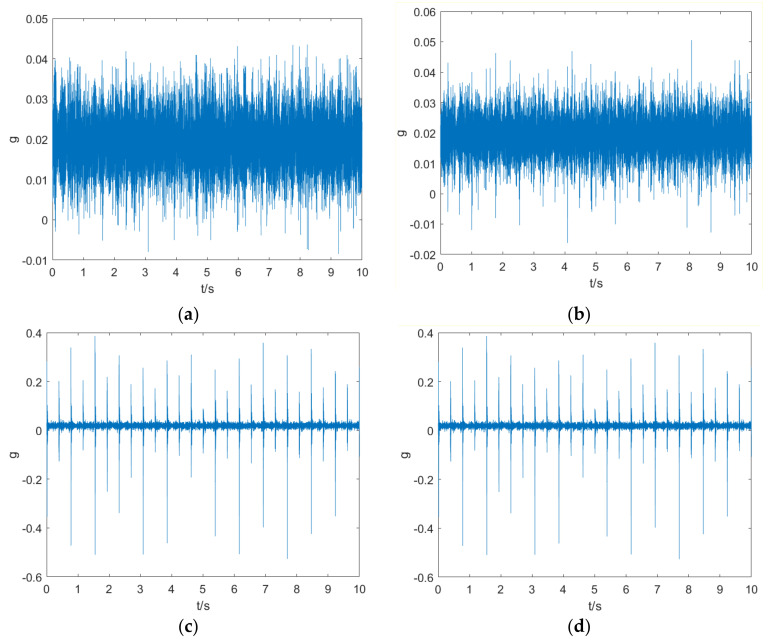
Samples of vibration signals in different conditions: (**a**) normal; (**b**) misalignment; (**c**) broken tooth; (**d**) combined fault.

The vibration signals in normal, misalignment, broken tooth and combined fault (misalignment and tooth broken) conditions were collected from the test rig. Set two measuring point, at the vertical and horizontal direction of the gearbox high-speed output shaft side, with a sampling frequency of 1 kHz and a sample time of 20 s. In the normal and broken tooth conditions, 18 sets of data were collected at the motor speed from 200 r/min to 720 r/min respectively. In misalignment condition, 26 sets of data were collected at the motor speed from 200 r/min to 680 r/min. In combined fault condition, 10 sets of data were collected at the motor speed from 200 r/min to 520 r/min. After preliminary frequency domain analysis of the signals, only the vertical direction signal is used for diagnosis in this paper. With non-overlapping 10,000 points of the signal, the samples in different conditions are shown in Figure 4, from which it can be seen that the presence of broken tooth is easy to distinguish, while the presence of misalignment is not.

### 3.2. LPFEWT and Comparison with Other Approaches

**Figure 5:**
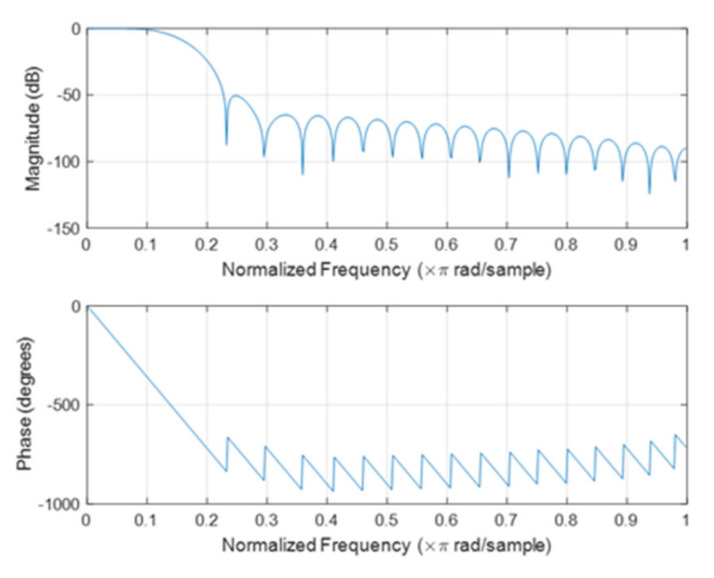
The magnitude and phase responses of the designed FIR low-pass filter.

**Figure 6:**
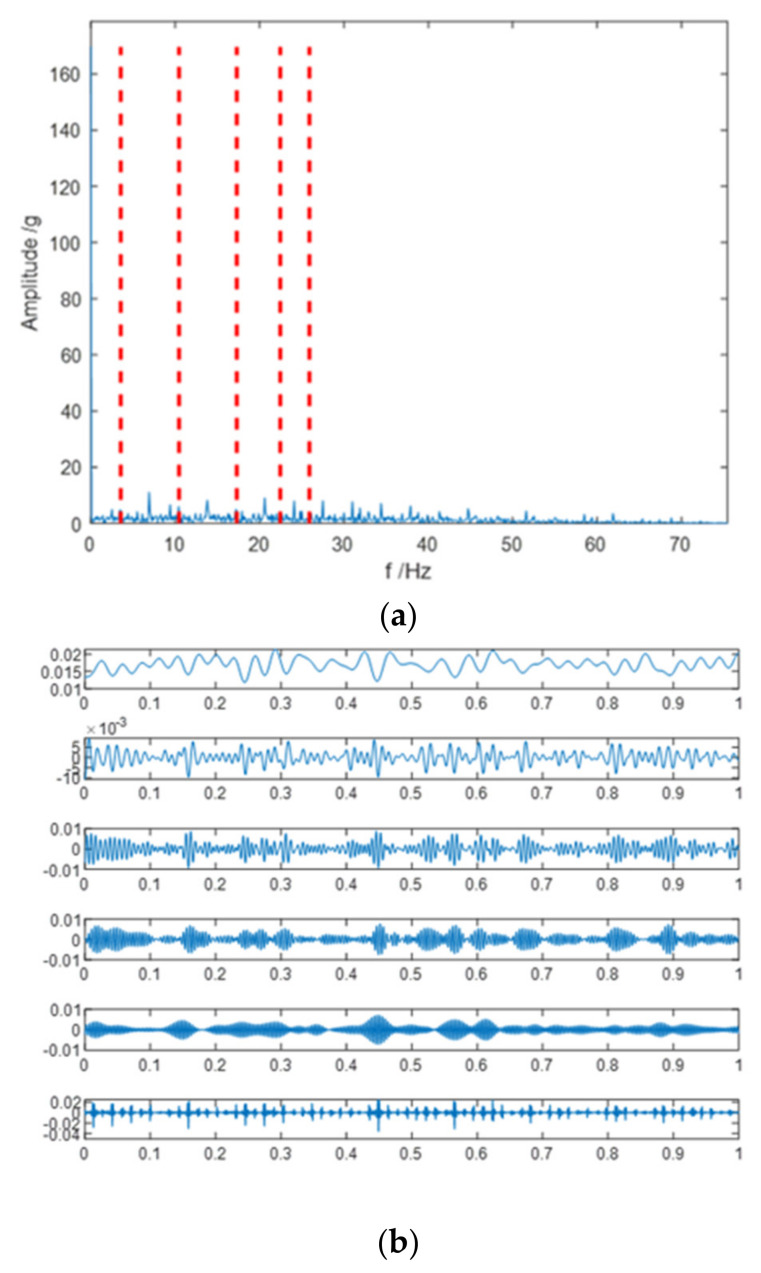
The EWT decomposition results of a combined fault signal: (**a**) Fourier spectrum segmentation; (**b**) empirical mode components.

Employ LPFEWT to extract features from the signal. The cut-off frequency of the low-pass filter is 50 Hz, about 6 times the rated rotating frequency of the generator. The magnitude and phase responses of the designed 40th-order Hamming Window FIR low-pass filter are shown in Figure 5. The filtered signal is decomposed by EWT and the number of EWT Fourier spectrum segments is set to 6. The EWT decomposition results of a combined fault signal are shown in Figure 6, obtained 6 empirical mode components from low frequency to high frequency. Discard the highest frequency component (the 6th mode) and calculate features of the left 5 empirical modes.

**Figure 7:**
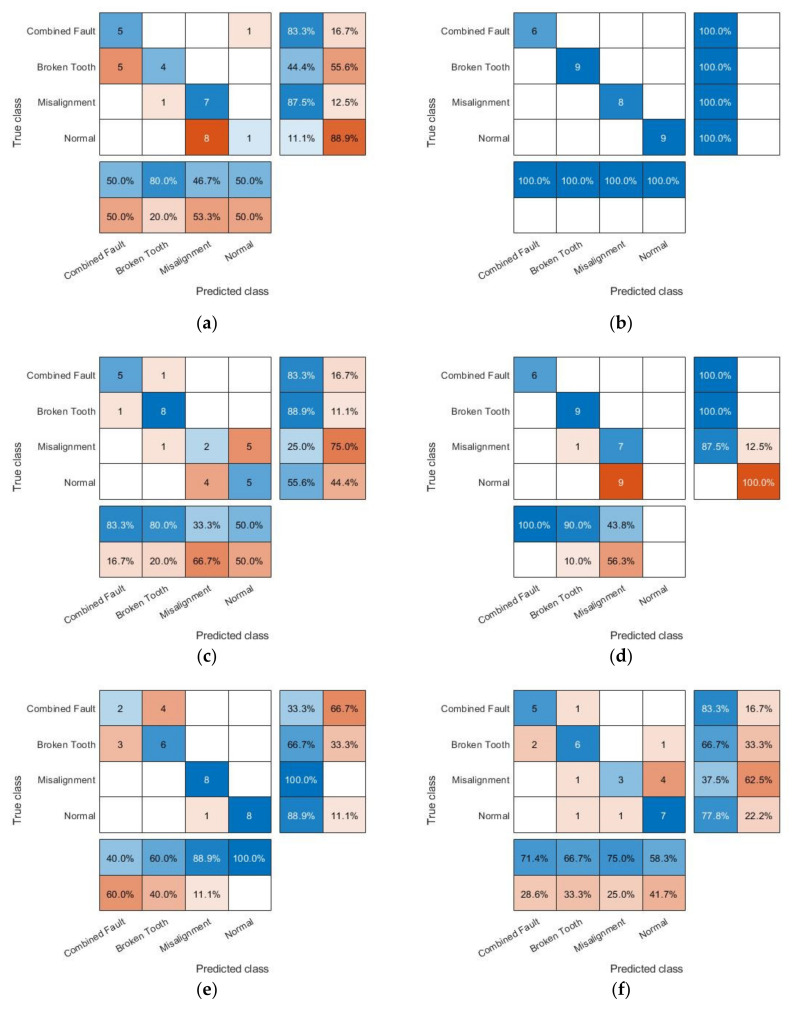
Confusion matrix charts of fault diagnosis results with features obtained by different approaches: (**a**) EWT; (**b**) LPFEWT; (**c**) EMD high frequency components; (**d**) LPFEMD high frequency components; (**e**) EMD low frequency components; (**f**) LPFEMD low frequency components.

We choose energies of the components as features, that is, the sum of the squares of the amplitude. There are 20 combined fault samples, 27 broken tooth samples, 26 misalignment samples and 27 normal samples, 100 samples in total. Shuffle the dataset and save. Take 14 combined fault samples, 18 broken tooth samples, 18 misalignment samples and 18 normal as training set. The remaining 32 samples of the dataset is testing set. We use LIBSVM Version 3.24 package for SVM classification under MATLAB 2018b. Train the SVM classification model for fault diagnosis, using GWO algorithm search the optimum values of penalty term C and RBF kernel parameter γ in the range of $\left[0.01,100\right]$. The average accuracy of 3-fold cross-validation of the training set is used as the fitness of the agents. The grey wolf population is set as 100 and the iteration is set as 50. Empirical modal decomposition (EMD) which is similar to EWT is chosen for comparison. Energies of components obtained by different approaches are inputs of the SVM model. Figure 7 shows the confusion matrix obtained by inputting the components energies of different methods into the SVM model. The horizontal direction represents the predicted class, and the vertical direction represents the true class. The 4×4 matrix is the number of samples of each type, and the percentage includes the prediction accuracy rate, false alarm rate and missing alarm rate of each type. Comparison of results are shown in Table 1. Different approaches with ‘LPF’ prefix use the same FIR low-pass. All approaches use same amounts of components of the signals.

From Figure 7 and Table 1 we can see, the testing set accuracy of using EWT directly is low, only 53.125%, and there is a lot of conditions confusions. Using LPFEWT to extract time-frequency domain features, the testing set accuracy is highly improved, reaching 100%. In addition, using EWT directly has high false alarm rate, while LPFEWT solves this problem. Among approaches based on EMD, EMD low frequency components has the highest accuracy and the lowest false alarm rate and missing alarm rate, which is 75%, but there are confusions between combined fault and broken tooth or misalignment and normal condition. LPFEMD low frequency components can only identify combined fault and broken tooth correctly. Both with or without the low-pass filter, EMD low frequency components has lower false alarm rate than the high frequency components. Both using high and low frequency components, the accuracy of LPFEMD is lower than that of EMD, and the false alarm rate is higher. The use of low-pass filter in diagnosis with approaches based on EMD will decrease the accuracy instead of increase that, and increase the false alarm rate. Among the six approaches of feature extraction, LPFEWT has the best performance.

We also tried SVM with linear kernel, the accuracy of training set and testing set are 82.4% and 87.5% respectively. So the classification of the dataset is a nonlinear problem, using RBF kernel is proper.

### 3.3. LPFEWT with Different Number of Fourier Spectrum Segments

To explore the effect of the number of LPFEWT Fourier spectrum segments on fault diagnosis results, the diagnosis was carried out with different number of Fourier spectrum segments, using energies of empirical modes as features, the results are shown in Table 2.

From Table 2, it can be seen that when the number of LPFEWT Fourier spectrum segments is small, although the testing set has good accuracy, the training set accuracy is slightly lower. When the number of LPFEWT Fourier spectrum segments is 5, 6, 7, 8, the diagnosis performance does not change. When the number of LPFEWT Fourier spectrum segments is 9, the accuracy of training set is improved a little, but the accuracy of testing set is reduced. Therefore, the number of LPFEWT Fourier spectrum segments should not be too small or too large, and there is a range of proper number of segments. It is suggested that the number of LPFEWT Fourier spectrum segments is set to 6 first, if the diagnosis results is not good enough, increase the number of segments one by one.

### 3.4. Effectiveness of the Proposed SVM Based Method

In the proposed method, we choose SVM for classification because it has superiority when dealing with small datasets. Since the samples of wind turbines in faults are relatively few. So deep learning which needs a large dataset is not suitable. Considering the speed of prediction after training, k-nearest neighbors (k-NN) algorithm which computes the distances between the instance and all the training instances to make decisions is abandoned. We compared SVM with naive Bayes, decision trees, random forests and artificial neural networks (ANN), the results are shown in Table 3.

From Table 3, we can see, for this classification problem, SVM has the best training performance and the accuracy of the training set is 94.1176%. The decision trees model has the lowest accuracy on training set with the highest accuracy as SVM model on testing set. All the models have good generalization ability. This show the feature selected is powerful. SVM has the best testing set accuracy and medium training set accuracy. Obviously, SVM is the best choice for this particular wind turbine fault diagnosis problem, which has good generalization ability even on a small dataset and easy to use (only has two hyperparameters need to tune).

## 4. Conclusions

This paper studies a ML-based fault diagnosis method for combined fault of wind turbines. LPFEWT is proposed to extract time-frequency domain features from vibration signals. And a GWO hyperparameter tuned SVM is employed for fault diagnosis. The method is verified on a DFIG wind turbine drivetrain fault test rig in the laboratory. The experimental results show that LPFEWT can greatly improve the accuracy of fault diagnosis and it is superior to other feature extraction approaches. The effect of the number of LPFEWT Fourier spectrum segments on fault diagnosis results is explored and a reasonable strategy to choose the number of segments is given. SVM is proved to be superior in this classification problem.

Compared with the existing analysis methods for combined fault, this ML-based method is efficient. After training the ML model at low computation costs, it can quickly handle the data of wind turbines working at different speeds and easily identify the faults without human knowledge. The method can also be applied to fault diagnosis of other rotating machinery.

## Figures and Tables

**Table 1:** Comparison of Fault Diagnosis Results with Different Feature Extraction Approaches.

Approach	C	γ	Training Set Accuracy	Testing Set Accuracy	False Alarm Rate	Missing Alarm Rate
EWT	98.135258	4.997962	80.8824% (55/68)	53.125% (17/32)	88.9% (8/9)	4.3% (1/23)
LPFEWT	66.953529	57.624745	94.1176% (64/68)	100% (32/32)	0% (0/9)	0% (0/23)
EMD high frequency components	17.297601	39.468164	76.4706% (52/68)	68.75% (22/32)	44.4% (4/9)	21.7% (5/23)
LPFEMD high frequency components	45.388002	96.255492	76.4706% (52/68)	62.5% (20/32)	100% (9/9)	0% (0/23)
EMD low frequency components	26.988942	37.129502	85.2941% (58/68)	75% (24/32)	11.1% (1/9)	0% (0/23)
LPFEMD low frequency components	48.145791	1.052425	69.1176% (47/68)	65.625% (21/32)	22.2% (2/9)	21.7% (5/23)

**Table 2:** Diagnosis Results of Employing LPFEWT with Different Number of Fourier Spectrum Segments.

Number of Segments	C	γ	Training Set Accuracy	Testing Set Accuracy
3	54.450584	44.708328	88.2353% (60/68)	100% (32/32)
4	43.410799	96.515668	92.6471% (63/68)	100% (32/32)
5	49.290038	78.087215	94.1176% (64/68)	100% (32/32)
6	66.953529	57.624745	94.1176% (64/68)	100% (32/32)
7	60.868225	95.642439	94.1176% (64/68)	100% (32/32)
8	98.149020	74.985752	94.1176% (64/68)	100% (32/32)
9	80.564115	91.484842	95.5882% (65/68)	96.875% (31/32)

**Table 3:** Comparison Results of Different ML Classification Models.

Model	Training Set Accuracy	Testing Set Accuracy	False Alarm Rate	Missing Alarm Rate
SVM	94.1176% (64/68)	100% (32/32)	0% (0/9)	0% (0/23)
Naive Bayes	95.5882% (65/68)	96.875% (31/32)	0% (0/9)	0% (0/23)
Decision trees	89.7059% (61/68)	100% (32/32)	0% (0/9)	0% (0/23)
Random forests	97.0588% (66/68)	96.875% (31/32)	0% (0/9)	0% (0/23)
ANN	92.6471% (63/68)	96.875% (31/32)	0% (0/9)	0% (0/23)
